# Protein Composition and Starch Structural Differentiation During Wheat Grain Filling Are Associated with Mature Grain Quality

**DOI:** 10.3390/foods15183340

**Published:** 2026-09-20

**Authors:** Hairong Wang, Zhiwei Sun, Fuyuan Deng, Yifan Du, Yongqing Wang, Na Niu, Lingjian Ma

**Affiliations:** College of Agronomy, Northwest A&F University, Yangling 712000, China; 2022060025@nwsuaf.edu.cn (H.W.); 15648591512@163.com (Z.S.); d1098515773@163.com (F.D.); 19163567376@163.com (Y.D.); 19871832979@163.com (Y.W.)

**Keywords:** cultivar variation, storage reserves, developmental dynamics, granule-size distribution, physicochemical properties, flour functionality

## Abstract

Wheat grain quality is progressively established through the accumulation and structural organization of storage reserves during grain filling, yet the relationships between developmental traits and mature-grain quality remain incompletely understood. This study aimed to characterize changes in grain developmental traits during grain filling and to clarify their relationships with mature-grain quality, with particular attention to quality attributes relevant to the use of wheat grain as a raw material for food production. In this study, four wheat cultivars contrasting in mature-grain quality were evaluated with mature-grain quality assessed over two harvest years and grain-filling dynamics examined during the 2024–2025 growing season. Grain growth was characterized at 10, 16, 23, and 29 days after anthesis (DAA), while protein and starch accumulation and protein fractions were examined during grain development and at maturity. Starch granule-size distribution, relative crystallinity, and thermal properties were further assessed at 23 and 29 DAA and at maturity, together with mature-flour pasting properties and correlations between developmental and mature-quality traits. All cultivars showed similar basic patterns of grain weight gain, dehydration, and progressive protein and starch accumulation, whereas cultivar-specific differences in protein fractions, amylose content, starch granule characteristics, and starch structure became increasingly evident during development. Although differences in total starch content among some cultivars narrowed at maturity, amylose content, granule size, B-type granule proportion, relative crystallinity, and thermal properties remained distinct and were associated with variation in gluten-related and pasting properties. Within the growing season examined, developmental traits at both 23 and 29 DAA were associated with mature-grain quality, with more significant associations detected at 29 DAA than at 23 DAA. These season-specific associations suggest that changes in protein composition and starch structure during mid-to-late grain filling may be related to mature-grain quality. These results help clarify how grain-filling characteristics are associated with final wheat grain quality.

## 1. Introduction

Wheat grain quality is a complex trait shaped by grain texture, protein content and composition, starch composition and structural characteristics, and the resulting processing properties. Grain hardness influences not only milling behavior but also the interfacial properties between starch granules and the protein matrix in the endosperm; grain-hardness-related proteins such as PINA and PINB contribute to grain texture by modulating starch–protein interfacial interactions [1,2]. Meanwhile, the contents, proportions, and polymerization characteristics of gliadins and glutenins contribute to gluten network formation, whereas amylose content, starch granule-size distribution, and structural organization further influence pasting and processing properties [3]. Therefore, mature-grain quality is not determined by any single protein or starch trait but rather represents an integrated phenotype arising from the combined effects of protein composition, starch composition and structure, and endosperm organization [4,5].

These mature-grain quality characteristics are progressively established during post-anthesis grain development. As the endosperm develops, carbon and nitrogen assimilates are continuously imported and converted into starch and storage proteins, while the deposition and organization of these storage reserves exhibit distinct temporal and spatial dynamics [6]. Wheat storage proteins undergo continuous synthesis, transport, and protein-body formation in the developing endosperm, and different classes of storage proteins show distinct deposition patterns [7,8]. Transcriptomic analyses have further revealed stage-specific expression of genes associated with storage-reserve deposition [9], while comparative studies of wheat cultivars with contrasting quality characteristics have demonstrated marked genotypic differences in protein and starch accumulation during grain filling [10]. Coordinated regulation of starch and storage-protein synthesis by transcription factors such as NAC-A18 further indicates that grain growth and storage-reserve accumulation are developmentally interconnected rather than independent processes [11].

Grain quality formation involves not only the accumulation of storage reserves but also changes in protein composition and starch structure. Gliadins and glutenins exhibit distinct accumulation patterns during grain development, and their deposition rates and relative proportions may vary with developmental stage [12]. Similarly, starch development involves more than an increase in total starch content: amylose content, amylopectin fine structure, granule composition, and crystalline organization may continue to change during grain filling [13,14]. These structural features are closely associated with starch gelatinization temperature, gelatinization enthalpy, and pasting behavior, indicating that mature starch functionality reflects the combined effects of multiple structural characteristics [15]. From a processing perspective, starch composition and structural organization also influence flour functionality and the quality of wheat-based products. Amylose content affects starch gelatinization and pasting properties [16], whereas starch granule size and starch–gluten interactions influence dough mixing and rheological behavior [17,18,19]. These starch-related properties also affect final product quality, including crumb texture and staling characteristics of bread [20], as well as the texture and cooking quality of wheat-based noodles [3]. Starch granule formation is also developmentally regulated. Wheat endosperm starch typically shows a bimodal granule-size distribution, with larger A-type granules initiated earlier and smaller B-type granules appearing later during grain development [21]. Recent genetic studies have identified several factors involved in the initiation and abundance of B-type starch granules [22,23], further supporting the developmental basis of the characteristic A/B-type granule distribution. Therefore, mature starch properties should be evaluated not only in terms of total starch content but also in relation to amylose content, granule-size distribution, crystalline organization, and other structural features.

Although previous studies have provided important insights into storage-protein deposition, starch biosynthesis, starch-granule initiation, and starch structural development, relatively few studies have integrated these trait levels to examine how wheat cultivars with contrasting mature-grain quality profiles differ during grain filling. In particular, it remains unclear at which developmental stages cultivar differences in protein composition and starch structural characteristics become more pronounced and how these developmental differences are associated with mature-grain quality. Accordingly, four wheat cultivars with contrasting mature-grain quality characteristics—QM725, FM8, WL169, and XN9198—were selected for this study. Mature-grain quality was evaluated across two harvest years, and the dynamics of grain growth, protein and starch accumulation, protein fractions, and starch granule and physicochemical characteristics were characterized during grain development. Associations between mid-to-late grain-filling traits and mature-grain quality were further analyzed. This study aimed to characterize shared and cultivar-specific developmental patterns of grain filling and to assess how differences in protein composition and starch structural development are associated with mature-grain quality, thereby providing a developmental basis for understanding cultivar differences in grain quality relevant to food use.

## 2. Materials and Methods

### 2.1. Plant Materials and Sample Collection

**Figure 1:**
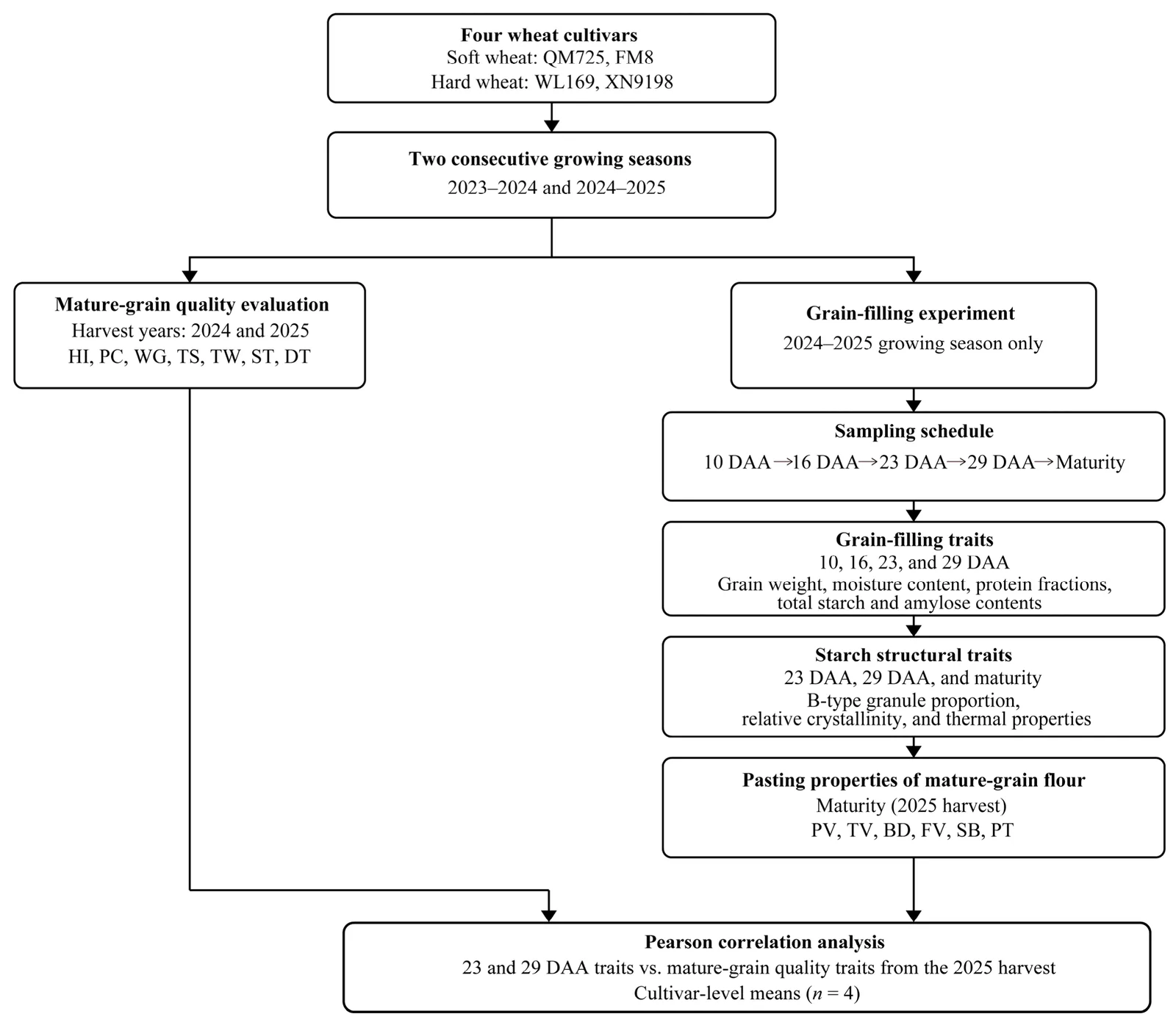
Schematic overview of the experimental design.

The four wheat cultivars were grown during two consecutive winter wheat growing seasons (2023–2024 and 2024–2025) at the experimental field of Northwest A&F University in Yangling, Shaanxi, China (34°18′ N, 108°1′ E; approximately 527 m above sea level), and mature grains were harvested in June 2024 and June 2025, respectively. The cultivars included two soft wheat cultivars, Quanmai 725 (QM725) and Fanmai 8 (FM8), and two hard wheat cultivars, Weilong 169 (WL169) and Xinong 9198 (XN9198). Mature-grain quality was evaluated in both harvest years, whereas grain-filling dynamics were examined only during the 2024–2025 growing season. A schematic overview of the experimental design and sampling strategy is presented in Figure 1.

For grain-filling sampling, plants with uniform growth and normal development were selected at anthesis, and spikes flowering on the same day were tagged. Normally developed grains from the middle portion of the tagged spikes were collected at 10, 16, 23, and 29 days after anthesis (DAA). Three biological replicates were included for each cultivar at each developmental stage. Samples used for fresh weight, grain morphology, and moisture content were analyzed immediately after collection, whereas those used for protein-fraction and starch analyses were processed and stored according to the corresponding analytical procedures.

### 2.2. Determination of Grain Quality Traits

According to AACC Method 55-31, the grain hardness index was determined using a Single Kernel Characterization System (SKCS 4100, Perten Instruments North America Inc., Springfield, IL, USA). Impurities, broken kernels, and visibly abnormal grains were removed before analysis. For each biological replicate, 300 intact kernels were randomly measured, and the result was expressed as hardness index (HI). Cultivars are commonly classified as soft wheat (HI < 45), mixed/medium-hard wheat (45 ≤ HI ≤ 60), or hard wheat (HI > 60).

Grain protein content, wet gluten content, and test weight were determined using a near-infrared analyzer (DA 7250, Perten Instruments AB, Hägersten, Sweden) [24]. Total starch and amylose contents were determined using commercial assay kits (Solarbio Science & Technology Co., Ltd., Beijing, China).

Dough development time (DT) and stability time (ST) were measured using a micro-doughLAB (Perten Instruments AB, Hägersten, Sweden), according to AACCI Method 54-70.01 [25].

### 2.3. Grain Morphological and Growth Measurements

Grains were collected at 10, 16, 23, and 29 DAA. For each biological replicate, 30 grains were randomly selected, and grain length, width, and thickness were measured using a digital vernier caliper. Additional grains were used to determine the hundred-grain fresh weight. The samples were then heated at 105 °C for 20 min and dried at 80 °C to constant weight to determine hundred-grain dry weight and calculate grain moisture content, with slight modifications to a previously described method [26]. Grain moisture content was calculated as follows:

Moisture content (%) = (Fresh weight − Dry weight)/Fresh weight × 100

### 2.4. Determination of Protein Fractions

Grain protein fractions were determined by sequential extraction based on Osborne solubility classification, with slight modifications to previously reported methods [27,28]. Freeze-dried grain powder (0.10 g) was sequentially extracted to obtain albumin, globulin, gliadin, and glutenin fractions. Briefly, 1.0 mL of distilled water was added to each sample, followed by shaking at 180 rpm for 30 min at room temperature and centrifugation at 4000 rpm for 15 min. The supernatant was collected, and the residue was extracted twice more under the same conditions. The three supernatants were pooled as the albumin extract. The remaining residue was then sequentially extracted with 1.0 mL of 10% (*w*/*v*) NaCl, 70% (*v*/*v*) ethanol, and 0.2% (*w*/*v*) NaOH under the same shaking and centrifugation conditions to obtain the globulin, gliadin, and glutenin fractions, respectively. Each fraction was extracted three times, and the corresponding supernatants were pooled. Protein concentrations in each fraction were determined using a BCA Protein Assay Kit (Solarbio Science & Technology Co., Ltd., Beijing, China) according to the manufacturer’s instructions [29]. Three biological replicates were analyzed for each cultivar at each developmental stage.

### 2.5. Starch Extraction and Particle-Size Analysis

Wheat flour or freeze-dried grain powder was mixed with distilled water to form a dough, which was washed repeatedly with distilled water to isolate crude starch. The resulting starch suspension was treated with 0.2% (*w*/*v*) NaOH for 4 h at room temperature and then centrifuged at 4000× *g* for 10 min. The upper layer containing impurities was removed, and the alkaline washing and centrifugation steps were repeated until no visible impurities remained. The starch was subsequently washed with absolute ethanol and centrifuged at 4000× *g* for 10 min at room temperature. The recovered starch was dried at 35 °C for 48 h, finely ground, passed through a 100-mesh sieve, and stored at 4 °C until further analysis [30]. Starch granule-size distribution was determined using a laser diffraction particle-size analyzer (SYNC, Microtrac Inc., York, PA, USA) with a wet-dispersion system using distilled water as the dispersant. The refractive indices of water and starch were set to 1.33 and 1.53, respectively. Samples collected at 23 and 29 DAA and at maturity were analyzed. Particle-size parameters, including D50, D[4,3], and D[3,2], were derived from the volume-based particle-size distribution. D50 denotes the volume median diameter, D[4,3] the volume-weighted mean diameter, and D[3,2] the surface-area-weighted mean diameter. The proportion of B-type starch granules was determined separately from the number-based particle-size distribution, and number-based distribution curves were plotted to show the relative numerical abundance of starch granules across different size classes [17]. Starch granules with diameters ≤ 10 μm were classified as B-type granules, whereas those with diameters > 10 μm were classified as A-type granules. The B-type granule number proportion (BNP) was calculated as the cumulative number percentage of starch granules with diameters ≤ 10 μm relative to the total detected granule number [31].

### 2.6. Starch Crystalline Structure

The crystalline structure of wheat starch was characterized using an X-ray diffractometer (D8 ADVANCE A25, Bruker AXS GmbH, Karlsruhe, Germany) at 40 kV and 100 mA [18]. Diffraction patterns were recorded over a 2θ range of 5–45° with a step size of 0.02° and a scanning rate of 5°/min. Relative crystallinity (RC) was calculated from the ratio of crystalline peak area to total diffraction area using JADE 9 (Materials Data Inc., Livermore, CA, USA). Measurements were performed on starch samples collected at 23 and 29 DAA and at maturity.

### 2.7. Starch Thermal Properties

The thermal properties of wheat starch were determined using differential scanning calorimetry (DSC; Q2000, TA Instruments, New Castle, DE, USA) [32]. Approximately 3 mg of starch was weighed into an aluminum pan and mixed with 9 μL of deionized water (starch-to-water mass ratio, approximately 1:3). The sealed pans were equilibrated at 4 °C for 20 h before analysis. An empty sealed pan was used as the reference, and samples were heated from 30 to 110 °C at 10 °C/min under a nitrogen flow of 50 mL/min. Onset temperature (T_o_), peak temperature (T_p_), conclusion temperature (T_c_), and gelatinization enthalpy (ΔH) were obtained using Universal Analysis 2000, version 4.5A (TA Instruments, New Castle, DE, USA). Measurements were performed on starch samples collected at 23 and 29 DAA and at maturity.

### 2.8. Pasting Properties of Mature Flour

The pasting properties of flour samples were determined using a Rapid Visco Analyzer (RVA 4500, Perten Instruments AB, Hägersten, Sweden) [33]. Samples were first held at 50 °C for 1 min, heated to 95 °C and held for 2.5 min, and then cooled to 50 °C and held for 2 min. Peak viscosity (PV), trough viscosity (TV), breakdown (BD), final viscosity (FV), setback (SB), and pasting temperature (PT) were recorded.

### 2.9. Statistical Analysis

All statistical analyses were performed using IBM SPSS Statistics 26.0 (IBM Corp., Armonk, NY, USA). For mature-grain quality traits measured in the two harvest years, two-way analysis of variance (ANOVA) was performed with cultivar and harvest year treated as fixed factors. The model included the main effects of cultivar and harvest year and their interaction (cultivar × harvest year). Three biological replicates for each cultivar × harvest-year combination were included in the analysis as independent observations. For grain-filling traits, two-way ANOVA was performed with cultivar and developmental stage as fixed factors, including the cultivar × developmental-stage interaction. Pearson correlation analysis was used to evaluate the relationships between grain developmental traits measured at 23 and 29 DAA and mature-grain quality traits from the corresponding 2024–2025 growing season (2025 harvest). Before correlation analysis, the three biological replicates for each trait were averaged within each cultivar, and the cultivar means were used as independent observations. Thus, each Pearson correlation coefficient was calculated using four cultivar-level observations (*n* = 4), with separate analyses conducted for 23 and 29 DAA. Origin 2024b (OriginLab Corporation, Northampton, MA, USA) was used for graphical presentation. All measurements were conducted with three biological replicates, and data are presented as mean ± standard deviation (SD).

## 3. Results

### 3.1. Mature-Grain Quality Characteristics

Significant differences in mature-grain quality were observed among the four wheat cultivars in both harvest years (Table 1). WL169 and XN9198 had significantly higher hardness index and wet gluten content than QM725 and FM8 in both years. WL169 had the highest hardness index in 2024, whereas XN9198 had the highest value in 2025. XN9198 had significantly higher protein content than the other cultivars in 2024; in 2025, it did not differ significantly from WL169 but was significantly higher than QM725 and FM8. XN9198 also had significantly lower total starch content than the other three cultivars in both years. Dough stability time followed the order XN9198 > WL169 > FM8 > QM725 in both years, whereas the cultivar ranking for dough development time differed between years. Two-way ANOVA revealed significant cultivar effects on all mature-grain quality traits (*p* < 0.001). Harvest year significantly affected several traits, particularly hardness index and dough stability time, and significant cultivar × year interactions were detected for most traits (*p* < 0.05; Table S1). These results indicate substantial differences in mature-grain quality among cultivars, with the magnitude of cultivar differences varying between the two harvest years for most traits.

### 3.2. Grain Growth and Morphological Development During Grain Filling

**Figure 2:**
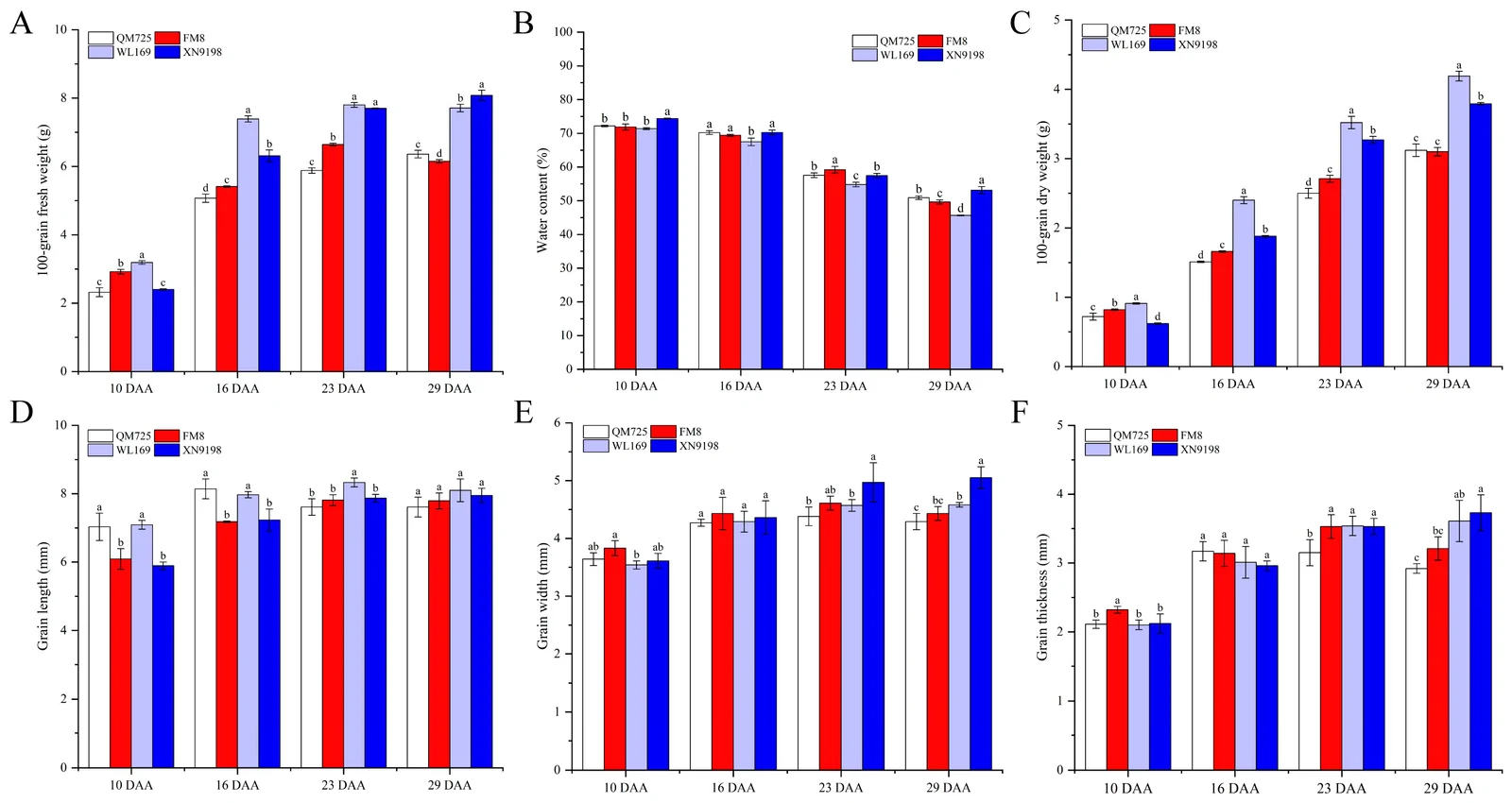
Grain growth dynamics of four wheat cultivars during grain filling. (**A**) Hundred-grain fresh weight; (**B**) grain moisture content; (**C**) hundred-grain dry weight; (**D**) grain length; (**E**) grain width; and (**F**) grain thickness at 10, 16, 23, and 29 days after anthesis (DAA). DAA, days after anthesis. Values are presented as mean ± standard deviation (SD). Different lowercase letters indicate significant differences among cultivars within the same developmental stage (*p* < 0.05).

Grain growth of the four wheat cultivars showed clear developmental-stage and cultivar-dependent differences at 10, 16, 23, and 29 days after anthesis (DAA) (Figure 2). Hundred-grain fresh and dry weights increased progressively during grain filling, whereas grain moisture content decreased (Figure 2A–C). WL169 consistently had the highest hundred-grain dry weight and maintained relatively high fresh weight from 10 to 23 DAA, whereas XN9198 showed pronounced late grain weight gain and had the highest fresh weight at 29 DAA. QM725 and FM8 generally exhibited lower fresh and dry weights during mid-to-late grain filling. Moisture loss also differed among cultivars, with XN9198 retaining the highest moisture content and WL169 the lowest at 29 DAA. Grain length increased rapidly from 10 to 16 DAA and changed only slightly thereafter, while grain width and thickness continued to change during mid-to-late grain filling, with cultivar-specific patterns (Figure 2D–F). XN9198 showed relatively greater grain width at 23 and 29 DAA and the greatest grain thickness at 29 DAA, whereas QM725 had relatively lower width and thickness at this stage. Considering the overall changes from 10 to 29 DAA, XN9198 showed the most pronounced increases in hundred-grain fresh weight, grain length, grain width, and grain thickness, whereas WL169 showed a relatively large increase in hundred-grain dry weight and a greater decline in grain moisture content. These results indicate that the magnitude of developmental change differed among cultivars and depended on the trait examined. Two-way ANOVA revealed significant effects of cultivar, developmental stage, and their interaction on hundred-grain fresh weight, hundred-grain dry weight, and moisture content (*p* < 0.001). Grain length and width were significantly affected by cultivar and developmental stage (*p* < 0.001), and significant cultivar × stage interactions were detected for all morphological traits (Table S2).

### 3.3. Protein and Starch Accumulation from Grain Filling to Maturity

**Figure 3:**
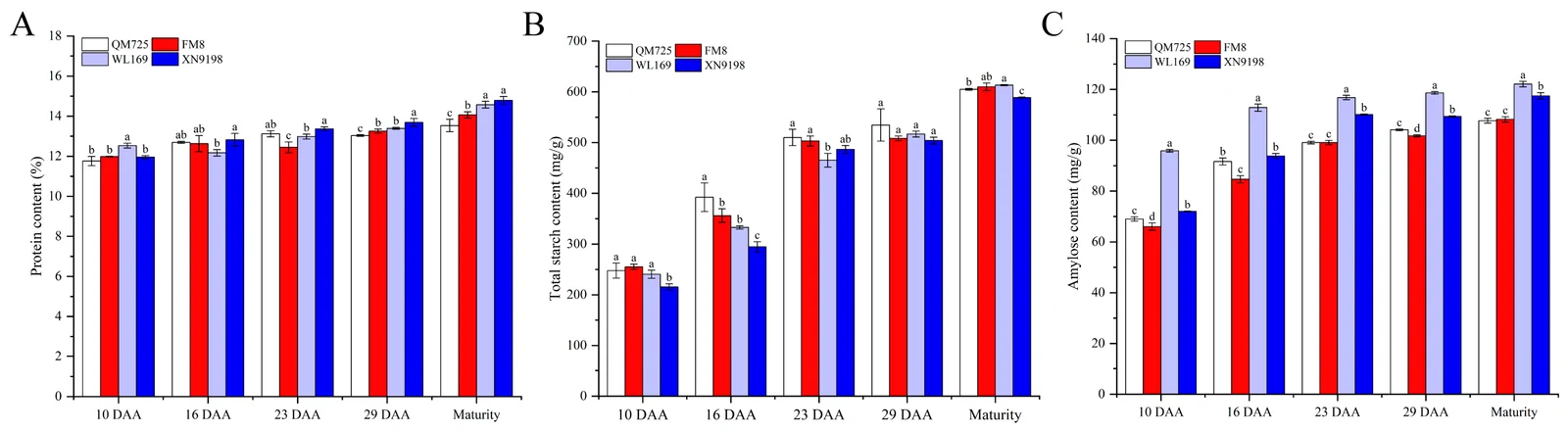
Dynamics of protein and starch accumulation in four wheat cultivars during grain filling and at maturity. (**A**) Protein content; (**B**) total starch content; and (**C**) amylose content at 10, 16, 23, and 29 DAA and at maturity. Values are presented as mean ± standard deviation (SD). Different lowercase letters indicate significant differences among cultivars within the same developmental stage (*p* < 0.05).

Protein, total starch, and amylose contents varied with developmental stage and cultivar (Figure 3). Protein content generally increased during grain filling, but the relative ranking of cultivars changed across stages. At 10 DAA, WL169 had a significantly higher protein content than the other three cultivars. By 29 DAA, XN9198 had the highest protein content and was significantly higher than that of the other cultivars. At maturity, WL169 and XN9198 had significantly higher protein contents than QM725, while FM8 showed an intermediate level (Figure 3A).

Total starch content increased markedly during grain filling, with the largest increase occurring from 10 to 23 DAA, followed by a slower increase toward maturity (Figure 3B). QM725 had a relatively high total starch content at the early developmental stages, particularly at 10 and 16 DAA. The differences among cultivars became less pronounced as grain filling progressed, although significant differences were still observed at 23 and 29 DAA. At maturity, FM8 showed an intermediate total starch content and did not differ significantly from either QM725 or WL169, whereas XN9198 had the lowest value.

Amylose content increased progressively from 10 DAA to maturity, while cultivar differences remained evident throughout grain filling (Figure 3C). WL169 consistently had the highest amylose content from 10 DAA to maturity, whereas XN9198 generally ranked second. QM725 and FM8 had lower amylose contents during most developmental stages, although their relative ranking varied at some stages. Thus, the cultivars differed not only in the amount of amylose accumulated, but also in the pattern of amylose accumulation during grain filling. Two-way ANOVA showed significant effects of cultivar, developmental stage, and their interaction on protein, total starch, and amylose contents (*p* < 0.001) (Table S3).

### 3.4. Protein Fraction Dynamics from Grain Filling to Maturity

**Figure 4:**
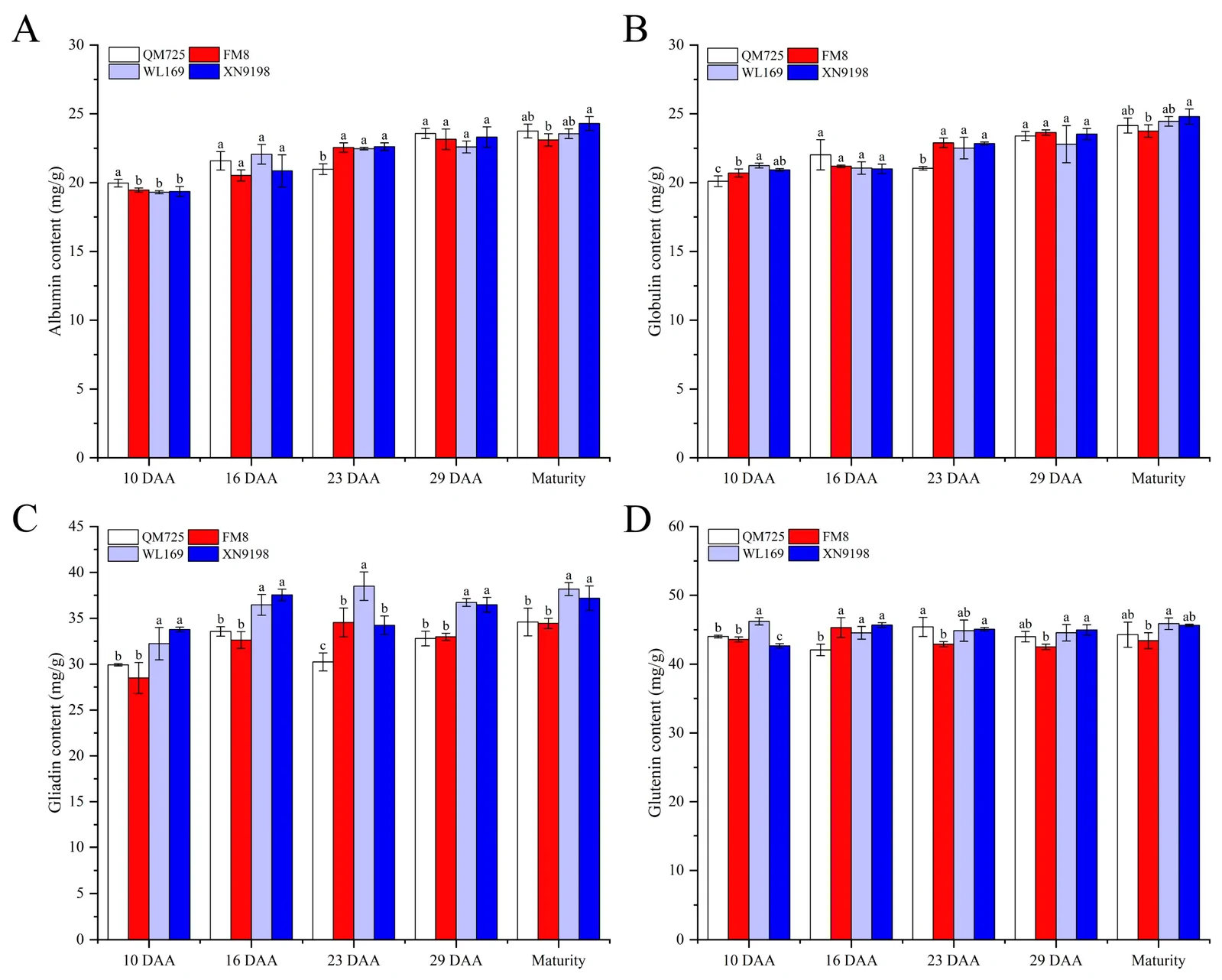
Dynamics of protein fractions in four wheat cultivars during grain filling and at maturity. (**A**) Albumin content; (**B**) globulin content; (**C**) gliadin content; and (**D**) glutenin content at 10, 16, 23, and 29 DAA and at maturity. Protein-fraction contents are expressed on a dry-weight basis (mg/g). Values are presented as mean ± standard deviation (SD). Different lowercase letters indicate significant differences among cultivars within the same developmental stage (*p* < 0.05).

The four protein fractions showed distinct developmental patterns and cultivar differences during grain development (Figure 4). Albumin content generally increased with development, although cultivar differences varied among stages. At maturity, XN9198 showed the highest mean albumin content, whereas FM8 had the lowest value (Figure 4A). Globulin content also generally increased during development. At 23 DAA, QM725 was significantly lower than the other cultivars, whereas cultivar differences were relatively small at 16 and 29 DAA (Figure 4B). Gliadin showed clearer cultivar differentiation. Its content generally increased during grain filling, and WL169 and XN9198 were significantly higher than QM725 and FM8 at 10 and 16 DAA. At 23 DAA, WL169 had the highest gliadin content and QM725 the lowest, while FM8 and XN9198 were intermediate. At 29 DAA and maturity, WL169 and XN9198 remained significantly higher than QM725 and FM8 (Figure 4C). Glutenin content changed relatively little overall, although significant cultivar differences occurred at some developmental stages (Figure 4D). Two-way ANOVA showed significant developmental-stage effects on albumin and globulin contents (*p* < 0.001), whereas their cultivar main effects were not significant (*p* > 0.05); significant cultivar × stage interactions were detected for both traits. Gliadin was significantly affected by cultivar, developmental stage, and their interaction (*p* < 0.001). Glutenin showed a significant cultivar effect and cultivar × stage interactions (*p* < 0.001), but no significant developmental-stage main effect (*p* > 0.05) (Table S3).

### 3.5. Starch Granule Characteristics from Mid-to-Late Grain Filling to Maturity

**Figure 5:**
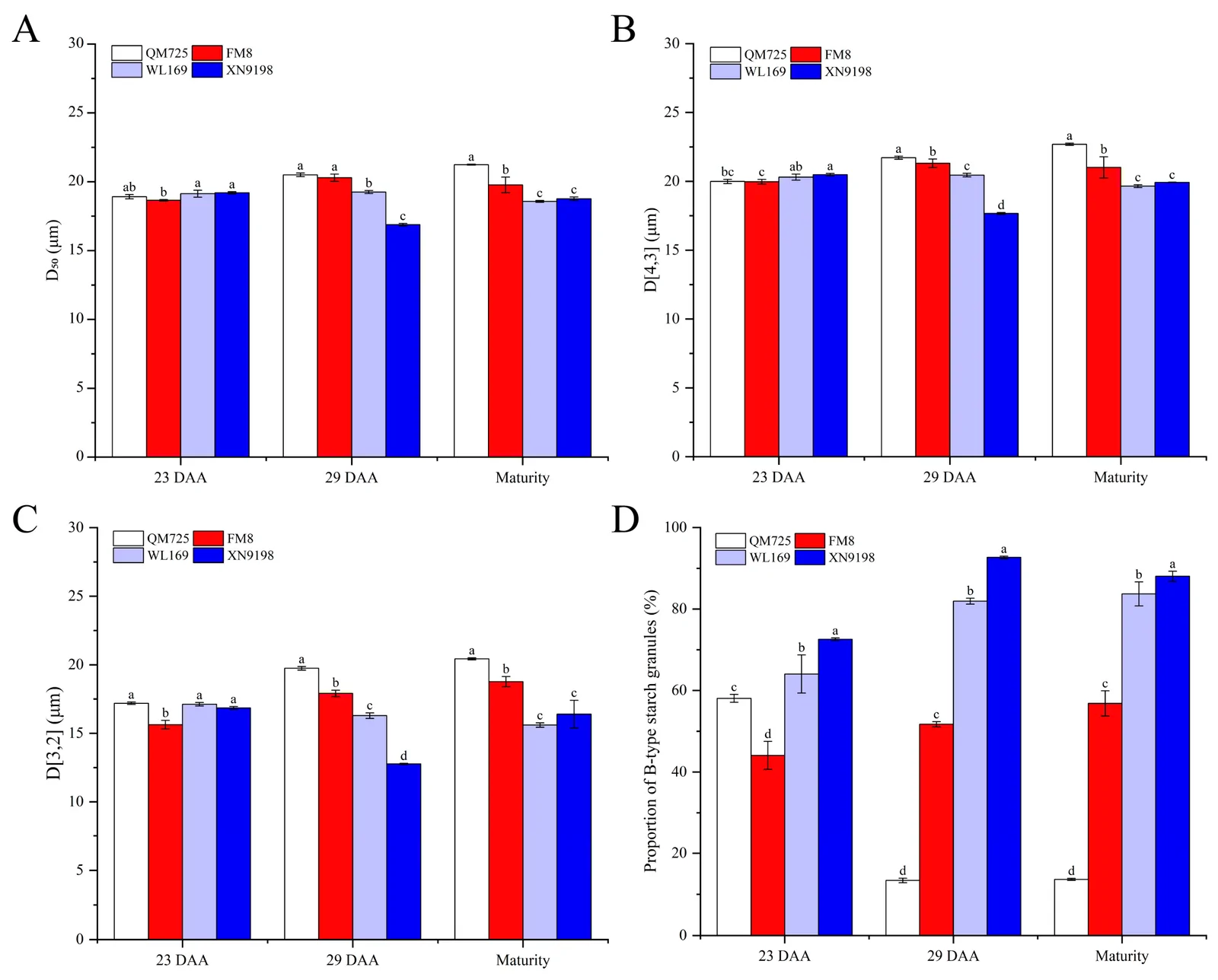
Changes in starch granule-size distribution of four wheat cultivars from late grain filling to maturity. (**A**) Median particle diameter (D50); (**B**) volume-weighted mean diameter (D[4,3]); (**C**) surface-area-weighted mean diameter (D[3,2]); and (**D**) B-type starch granule number proportion at 23 and 29 DAA and at maturity. Values are presented as mean ± standard deviation (SD). Different lowercase letters indicate significant differences among cultivars within the same developmental stage (*p* < 0.05).

Starch particle-size distribution showed clear cultivar differences from 23 DAA to maturity (Figure 5). Cultivar differences in D50, D[4,3], and D[3,2] were already significant at 23 DAA and became more pronounced at 29 DAA and maturity. At 29 DAA, QM725 generally showed relatively high D50, D[4,3], and D[3,2] values, whereas XN9198 had the lowest values. At maturity, all three particle-size parameters of QM725 were significantly higher than those of WL169 and XN9198, with FM8 generally intermediate (Figure 5A–C). The number-based particle-size distribution curves of the four cultivars at 23 and 29 DAA and at maturity are presented in Figure S1. The B-type starch granule number proportion showed stronger cultivar differentiation (Figure 5D). At 23 DAA, XN9198 had the highest proportion, followed by WL169, whereas FM8 had the lowest. At 29 DAA, the B-type granule proportions of XN9198 and WL169 reached approximately 93% and 82%, respectively, and were significantly higher than those of FM8 and QM725, while QM725 decreased to approximately 13%. At maturity, the order XN9198 > WL169 > FM8 > QM725 was maintained. Two-way ANOVA showed significant effects of cultivar, developmental stage, and their interaction on D50, D[4,3], and D[3,2] (*p* < 0.001). B-type granule number proportion was significantly affected by cultivar and cultivar × stage interactions (*p* < 0.001), whereas the stage main effect was not significant (*p* > 0.05) (Table S4).

### 3.6. Starch Crystalline Structure and Thermal Properties from Mid-to-Late Grain Filling to Maturity

The relative crystallinity and thermal properties of starch showed clear developmental-stage and cultivar differences (Table 2). All starch samples exhibited diffraction peaks at approximately 15°, 17°, 18°, and 23° (Figure S2). At 23 DAA, QM725 and XN9198 had relatively high relative crystallinity (RC) values of 32.20% and 32.02%, respectively, and both were significantly higher than FM8, which showed the lowest value (27.28%). At 29 DAA, QM725 had the highest RC (34.29%) and was significantly higher than the other cultivars. At maturity, XN9198 had the highest RC (32.83%), whereas QM725 showed the lowest value (29.93%).

DSC thermograms are shown in Figure S3. At 23 DAA, FM8 had the highest onset temperature (T_o_) and peak temperature (T_p_), whereas QM725 showed the highest values at 29 DAA. At maturity, T_o_ decreased to 55.86–57.16 °C, with no significant differences among cultivars. T_p_ also decreased overall, but significant cultivar differences remained, with WL169 showing the highest value and XN9198 the lowest. Conclusion temperature (T_c_) remained relatively stable, mainly within 73–75 °C, and no significant cultivar differences were detected at maturity. Gelatinization enthalpy (ΔH) also differed among cultivars and developmental stages, with XN9198 showing the highest value at 23 and 29 DAA and QM725 the lowest value at maturity. Two-way ANOVA showed significant effects of cultivar and developmental stage on RC, T_o_, T_p_, and ΔH (*p* < 0.001), together with significant cultivar × stage interaction. In contrast, T_c_ showed a significant cultivar effect (*p* < 0.01), whereas the effects of developmental stage and cultivar × stage interactions were not significant (*p* > 0.05) (Table S4).

### 3.7. Pasting Properties of Mature-Grain Flour

RVA parameters differed significantly among the four wheat cultivars (Table 3). FM8 and QM725 showed the highest peak viscosity (PV), with no significant difference between them, and both were significantly higher than WL169 and XN9198. XN9198 showed the lowest PV. Trough viscosity (TV) was highest in FM8 and significantly higher than in the other cultivars, whereas WL169 had the lowest value. Final viscosity (FV) was also highest in FM8 and lowest in WL169, while QM725 and XN9198 did not differ significantly. Breakdown (BD) differed significantly among all four cultivars, following the order QM725 > FM8 > WL169 > XN9198. Setback (SB) was relatively high in FM8 and XN9198, with no significant difference between them. Although pasting temperature (PT) varied within a relatively narrow range (86.43–87.71 °C), significant differences among cultivars were detected. XN9198 had the highest numerical PT, although it did not differ significantly from FM8, whereas WL169 showed the lowest PT.

### 3.8. Associations Between Grain-Filling Traits and Mature-Grain Quality

**Figure 6:**
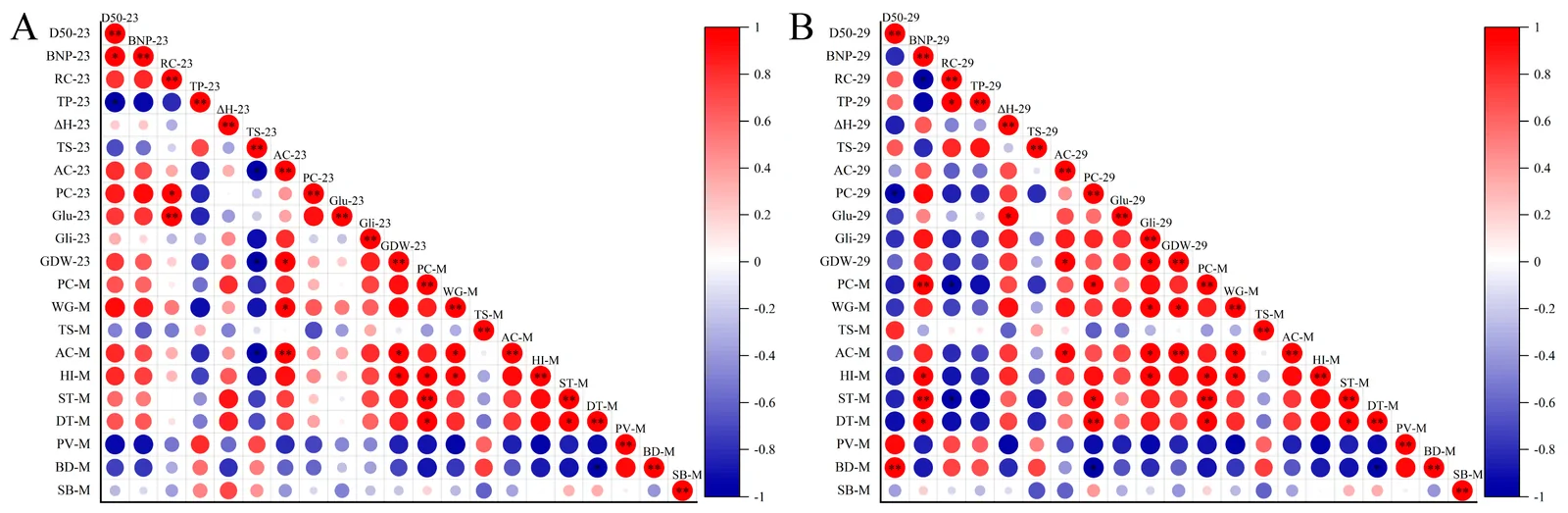
Correlations between grain developmental traits and mature-grain quality traits in four wheat cultivars. (**A**) Pearson correlation coefficients between grain developmental traits at 23 DAA and mature-grain quality traits; (**B**) Pearson correlation coefficients between grain developmental traits at 29 DAA and mature-grain quality traits. Colors indicate the direction and magnitude of the correlation coefficients, and circle size is proportional to the absolute value of the correlation coefficient. * and ** indicate significance at *p* < 0.05 and *p* < 0.01, respectively. GDW, hundred-grain dry weight; PC, protein content; Gli, gliadin content; Glu, glutenin content; TS, total starch content; AC, amylose content; D50, volume median diameter; BNP, B-type starch granule number proportion; RC, relative crystallinity; ΔH, gelatinization enthalpy; HI, hardness index; WG, wet gluten content; ST, dough stability time; DT, dough development time; PV, peak viscosity; BD, breakdown; SB, setback; M, maturity. Pearson correlation analysis was performed using cultivar-level means as independent observations (*n* = 4). Grain developmental traits at 23 and 29 DAA were correlated separately with mature-grain quality traits from the corresponding 2024–2025 growing season (2025 harvest).

Correlation analysis identified significant associations between grain developmental traits at both 23 and 29 DAA and mature-grain quality, with more significant correlations detected at 29 DAA (Figure 6). At 23 DAA, amylose content (AC) was positively correlated with mature-grain wet gluten content (WG) and AC, total starch content (TS) was negatively correlated with mature-grain AC, and grain dry weight (GDW) was positively correlated with mature-grain AC and hardness index (HI) (Figure 6A). At 29 DAA, B-type starch granule number proportion (BNP) was positively correlated with mature-grain protein content (PC), HI, dough stability time (ST), and dough development time (DT), while relative crystallinity (RC) was negatively correlated with PC and ST. Protein content and gliadin content at 29 DAA were also associated with several mature protein-, gluten-, and dough-related traits, whereas associations with pasting properties were limited; PC was negatively correlated with breakdown (BD), and D50 was positively correlated with BD. No significant associations with peak viscosity (PV) or setback (SB) were detected (Figure 6B).

## 4. Discussion

### 4.1. Cultivar-Specific Grain-Filling Patterns Associated with Mature-Grain Quality Differences

The four wheat cultivars exhibited distinct combinations of mature-grain quality traits, indicating that differences in grain texture, protein- and gluten-related characteristics, and starch pasting behavior were not always consistent across cultivars. This reflects the multidimensional nature of wheat quality, which depends on the combined contributions of protein, starch, and grain physical characteristics rather than on a single trait. Despite these mature-quality differences, all four cultivars showed broadly similar developmental patterns during grain filling, including progressive grain weight gain, dehydration, and storage-reserve accumulation. Grain length increased mainly during early-to-mid filling, whereas grain width and thickness continued to increase during later stages. Similar stage-dependent changes in starch and storage-protein deposition have been reported previously [34,35,36], supporting the view that the general developmental patterns observed in the present study are consistent with the typical progression of wheat grain filling.

Within this broadly shared developmental pattern, however, the cultivars exhibited different trajectories of storage-reserve accumulation and composition. WL169 generally maintained relatively high dry-matter and amylose levels, XN9198 showed relatively high protein content during late filling, whereas QM725 maintained relatively high total starch content. The significant cultivar × developmental stage interactions observed for most traits further indicate that cultivar differences changed across grain-filling stages rather than remaining constant throughout development. Previous studies have also shown that protein, glutenin macropolymer, amylose, and amylopectin accumulation are differentially regulated during grain development [34], while post-anthesis temperature, source–sink relationships, and carbon and nitrogen availability can further modify storage-reserve deposition and final grain quality [37,38]. Thus, differences in mature-grain quality were associated with cultivar-specific trajectories of storage-reserve accumulation and composition during grain filling.

### 4.2. Protein Fraction Dynamics and Their Associations with Mature Gluten-Related Quality

All four cultivars showed an overall increase in protein content during grain filling, whereas the individual protein fractions exhibited distinct developmental patterns. Albumin and globulin were significantly affected by developmental stage but not by the cultivar main effect, indicating a strong overall effect of grain development. However, the significant cultivar × stage interactions for both fractions showed that their temporal patterns differed among cultivars. Gliadin, in contrast, was significantly affected by cultivar, developmental stage, and their interaction, with cultivar differences becoming more evident during mid-to-late grain filling. Glutenin showed no significant stage main effect but significant cultivar and cultivar × stage effects, indicating relatively limited overall changes across developmental stages together with cultivar-dependent temporal variation. Previous studies have similarly shown that storage-protein deposition changes dynamically during wheat grain development and is influenced by genotype as well as by post-anthesis carbon and nitrogen supply [38,39].

Cultivars with longer dough stability times did not consistently show the highest glutenin content during grain development, suggesting that total glutenin content alone is insufficient to explain mature gluten-related properties. Gluten characteristics depend not only on the relative abundance of gliadins and glutenins but also on the Gli/Glu ratio, the composition of high- and low-molecular-weight glutenin subunits (HMW-GS and LMW-GS), glutenin polymerization, and intermolecular interactions. Previous studies have shown that reduced HMW-GS abundance can decrease glutenin macropolymer (GMP) accumulation and weaken the gluten network [40], while differences in protein composition, disulfide bonding, and secondary structure are also associated with dough and processing properties [41]. Wheat genotypes with contrasting end-use quality have also been reported to differ in storage-protein composition and accumulation patterns [42], and glutenin subunit composition is closely associated with wet gluten content, dough stability, and baking quality [43]. Therefore, the differences in mature gluten-related quality observed among the four cultivars likely reflect the combined contributions of total protein accumulation, storage-protein composition, and their developmental patterns rather than variation in any single protein fraction.

### 4.3. Starch Granule and Structural Development in Relation to Mature Pasting Properties

Total starch content increased during grain filling in all four cultivars, and differences among QM725, FM8, and WL169 narrowed markedly by maturity. In contrast, amylose content, starch granule-size distribution, B-type granule proportion, relative crystallinity, and thermal properties continued to differ among cultivars. These results indicate that similar final total starch contents do not necessarily correspond to similar starch composition or structural organization. This contrast was particularly evident between QM725 and XN9198: during late grain filling, QM725 was characterized by larger D50, D[4,3], and D[3,2] values and a lower proportion of B-type granules, whereas XN9198 showed a higher B-type granule proportion and smaller granule-size parameters. FM8 and WL169 exhibited different patterns of granule allocation and amylose accumulation, further indicating that distinct mature starch characteristics were associated with different developmental trajectories. A- and B-type starch granules differ in granule size, molecular composition, semicrystalline organization, and functional properties [44,45,46]. Variation at MRC-related loci can alter B-type granule abundance, A-type granule size, and RVA characteristics [47], while reductions in B-type granules can substantially modify starch physicochemical properties even when total starch content changes little [48]. Grain hardness in common wheat is closely associated with interactions between starch granules and the surrounding protein matrix [49]. In the present study, starch granule characteristics showed some correspondence with grain texture: the soft wheat QM725 had larger starch granules and a lower proportion of B-type granules, whereas the hard wheat XN9198 showed the opposite pattern. A similar directional trend was also observed for FM8 and WL169, particularly in B-type granule proportion, although differences in granule-size parameters were less pronounced. This is broadly consistent with previous reports of differences in A- and B-type starch granule proportions and physicochemical properties between soft and hard wheats [50]. Chichti et al. [1] using near-isogenic wheat lines differing in grain hardness, demonstrated distinct nanomechanical properties at the starch–protein interface between soft- and hard-textured wheat. In light of these findings, the association observed here between starch granule characteristics and grain hardness may be related to cultivar differences in endosperm organization and interactions between starch granules and the surrounding protein matrix, rather than to granule-size distribution alone.

The RC and DSC results further showed that starch structural development followed cultivar-dependent patterns from mid-to-late grain filling to maturity. RC, T_o_, and T_p_ changed differently among cultivars, whereas T_c_ remained comparatively stable. The significant cultivar × stage interactions detected for several traits further indicated that starch structural changes during development differed among cultivars. Previous studies have similarly shown that total starch deposition, amylose and amylopectin structural development, and crystalline organization follow partly distinct temporal patterns in wheat grain [36,51]. Importantly, RVA measurements in the present study were performed on flour, whereas particle size, XRD, and DSC analyses primarily characterized isolated starch. Flour pasting behavior therefore represents an integrated property influenced not only by starch characteristics but also by the surrounding protein matrix. Studies of high-amylose wheat have shown that RVA properties can be jointly influenced by amylose and amylopectin structure and protein composition [16]. In addition, variations in the Gli/Glu ratio and B-type granule proportion have been reported to influence starch swelling, amylose leaching, rheological behavior, and starch–gluten interactions during heating [19,52]. These factors may help explain why individual RVA parameters did not correspond consistently to any single starch structural trait in the present study. Overall, cultivar differences in mature pasting properties appear to reflect the combined influence of starch composition, granule allocation, structural organization, and the surrounding protein matrix rather than final total starch content alone.

### 4.4. Associations Between Mid-to-Late Grain-Filling Traits and Mature Quality

Significant associations between developmental traits and mature-grain quality were detected at both 23 and 29 DAA, with more significant correlations observed at 29 DAA. At the later stage, B-type granule proportion and protein-related traits were mainly associated with mature protein, gluten, grain hardness, and dough properties, whereas associations with mature pasting properties were more limited and trait-specific. These results indicate that mature quality is related to several aspects of storage-reserve accumulation and starch granule development rather than to any single developmental trait. This pattern is consistent with the developmental nature of wheat quality formation. Previous studies have shown that protein and carbohydrate metabolism vary markedly across grain-filling stages [10], while starch accumulation and structural development are not fully synchronized [36]. Although substantial starch deposition occurs relatively early, starch molecular organization and granule characteristics continue to change during subsequent development. In particular, B-type granules are initiated later than A-type granules, and variation in their abundance can influence final granule-size distribution and starch properties [23]. These developmental characteristics may help explain why associations between grain-filling traits and mature quality were more apparent at 29 DAA.

The four cultivars differed clearly in grain texture and quality, providing a useful basis for comparing cultivar-dependent patterns of grain development and mature quality. Evaluation over two consecutive harvest years also allowed year-to-year variation in mature quality to be examined under the conditions of this study. However, wheat quality is also influenced by environmental conditions and genotype × environment interactions [53]. Because grain-filling dynamics were assessed only during the 2024–2025 growing season, the developmental–quality associations identified here should be interpreted within the environmental context of that season, and their stability across years and environments requires further evaluation.

More significant associations with mature-grain quality were detected at 29 DAA than at 23 DAA, suggesting that late grain filling may be a more informative stage for examining cultivar-specific quality development under the conditions of this study.

## 5. Conclusions

The four wheat cultivars shared a broadly similar developmental pattern during grain filling, characterized by progressive grain weight gain, dehydration, and storage-reserve accumulation, while differing in the magnitude and timing of changes in protein composition and starch structure. Cultivar differences in protein fractions, amylose content, starch granule-size distribution, B-type granule proportion, relative crystallinity, and thermal properties became more evident during mid-to-late grain filling. Although differences in total starch content among some cultivars narrowed at maturity, compositional and structural differences persisted and were associated with mature gluten-related and pasting properties. Developmental traits at both 23 and 29 DAA were associated with mature-grain quality, with more significant associations observed at 29 DAA. These season-specific relationships suggest that mature-grain quality may be linked not only to final storage-reserve contents but also to the developmental dynamics of protein composition and starch structure during grain filling. These findings provide a developmental perspective for understanding wheat quality differentiation and for further evaluating late-filling traits associated with mature-grain quality.

## Figures and Tables

**Table 1:** Mature grain and dough quality traits of four wheat cultivars in two harvest years.

Trait	Harvest Year	QM725	FM8	WL169	XN9198
HI	2024	30.01 ± 0.95 f	34.18 ± 0.75 e	72.44 ± 0.77 a	62.84 ± 0.65 c
HI	2025	33.42 ± 0.42 e	41.02 ± 0.70 d	69.11 ± 0.50 b	71.84 ± 0.35 a
PC (%)	2024	13.49 ± 0.26 d	14.19 ± 0.43 c	14.43 ± 0.46 bc	15.31 ± 0.32 a
PC (%)	2025	13.53 ± 0.31 d	14.06 ± 0.16 c	14.57 ± 0.17 bc	14.79 ± 0.19 ab
WG (%)	2024	29.74 ± 0.31 c	28.27 ± 0.22 d	32.88 ± 0.64 a	32.56 ± 0.28 a
WG (%)	2025	29.76 ± 0.07 c	29.44 ± 0.28 c	31.47 ± 0.64 b	31.35 ± 0.47 b
TS (%)	2024	61.31 ± 0.37 ab	61.93 ± 0.31 a	61.43 ± 0.33 ab	58.63 ± 0.19 d
TS (%)	2025	60.49 ± 0.18 c	60.99 ± 0.73 bc	61.32 ± 0.12 ab	58.88 ± 0.10 d
TW (g/L)	2024	792.09 ± 1.02 d	796.52 ± 1.20 cd	803.43 ± 1.35 b	802.99 ± 1.83 bc
TW (g/L)	2025	774.76 ± 7.76 e	799.89 ± 0.63 bc	811.66 ± 1.20 a	797.91 ± 5.86 bcd
ST (min)	2024	1.55 ± 0.15 e	6.19 ± 0.27 c	8.60 ± 0.10 b	9.80 ± 0.60 a
ST (min)	2025	1.25 ± 0.05 e	4.80 ± 0.40 d	6.65 ± 0.55 c	8.20 ± 0.10 b
DT (min)	2024	2.10 ± 0.40 f	6.54 ± 0.41 b	4.85 ± 0.75 d	5.60 ± 0.10 c
DT (min)	2025	1.40 ± 0.01 f	3.80 ± 0.40 e	5.65 ± 0.05 c	7.80 ± 0.60 a

Note: HI, hardness index; PC, protein content; WG, wet gluten content; TS, total starch content; TW, test weight; ST, stability time; DT, dough development time. Values are presented as mean ± standard deviation (SD). Different lowercase letters within the same trait indicate significant differences among cultivar–harvest year combinations (*p* < 0.05).

**Table 2:** Relative crystallinity and thermal properties of starch from four wheat cultivars at 23 and 29 DAA and at maturity.

Parameter	Stage	QM725	FM8	WL169	XN9198
RC (%)	23 DAA	32.20 ± 0.61 a	27.28 ± 0.47 c	30.87 ± 0.36 b	32.02 ± 0.18 a
RC (%)	29 DAA	34.29 ± 0.47 a	30.98 ± 0.62 b	29.35 ± 0.11 c	29.61 ± 0.81 c
RC (%)	Maturity	29.93 ± 0.12 c	32.63 ± 0.48 ab	31.97 ± 0.58 b	32.83 ± 0.26 a
T_o_ (°C)	23 DAA	61.07 ± 0.22 b	62.51 ± 0.01 a	60.91 ± 0.32 bc	60.59 ± 0.12 c
T_o_ (°C)	29 DAA	62.47 ± 0.07 a	61.37 ± 0.16 b	60.78 ± 0.11 c	60.66 ± 0.05 c
T_o_ (°C)	Maturity	56.86 ± 0.52 a	57.16 ± 0.03 a	56.45 ± 1.26 a	55.86 ± 0.12 a
T_p_ (°C)	23 DAA	64.78 ± 0.43 b	66.12 ± 0.06 a	63.88 ± 0.07 c	64.08 ± 0.17 c
T_p_ (°C)	29 DAA	65.52 ± 0.47 a	63.97 ± 0.17 b	63.62 ± 0.03 b	63.63 ± 0.02 b
T_p_ (°C)	Maturity	61.11 ± 0.18 b	61.39 ± 0.21 b	61.83 ± 0.25 a	59.99 ± 0.15 c
T_c_ (°C)	23 DAA	73.07 ± 0.42 c	73.77 ± 0.30 b	74.24 ± 0.10 ab	74.42 ± 0.06 a
T_c_ (°C)	29 DAA	73.40 ± 0.13 b	74.12 ± 0.53 ab	74.77 ± 0.43 a	74.41 ± 0.47 a
T_c_ (°C)	Maturity	73.06 ± 0.81 a	74.17 ± 1.32 a	73.92 ± 0.50 a	73.87 ± 0.83 a
ΔH (J/g)	23 DAA	9.73 ± 0.08 c	10.07 ± 0.11 ab	9.96 ± 0.13 b	10.19 ± 0.13 a
ΔH (J/g)	29 DAA	9.87 ± 0.37 bc	9.45 ± 0.17 c	10.22 ± 0.22 ab	10.50 ± 0.24 a
ΔH (J/g)	Maturity	8.84 ± 0.30 b	10.02 ± 0.38 a	9.45 ± 0.04 ab	9.86 ± 0.45 a

Note: RC, relative crystallinity; T_o_, onset gelatinization temperature; T_p_, peak gelatinization temperature; T_c_, conclusion gelatinization temperature; ΔH, gelatinization enthalpy. Values are presented as mean ± standard deviation (SD). Different lowercase letters within the same developmental stage indicate significant differences among cultivars (*p* < 0.05).

**Table 3:** Pasting properties of flour from mature grains of four wheat cultivars.

Trait	QM725	FM8	WL169	XN9198
PV (cP)	2134.67 ± 76.17 a	2180.33 ± 43.02 a	1786.67 ± 71.11 b	1587.67 ± 44.00 c
TV (cP)	1123.67 ± 26.10 b	1254.67 ± 10.26 a	988.67 ± 64.00 c	1084.33 ± 65.84 b
BD (cP)	1011.00 ± 51.42 a	925.67 ± 40.72 b	798.00 ± 7.81 c	503.33 ± 61.85 d
FV (cP)	2042.67 ± 40.45 b	2260.33 ± 30.24 a	1867.67 ± 68.60 c	2094.33 ± 35.08 b
SB (cP)	919.00 ± 19.31 b	1005.67 ± 40.45 a	879.00 ± 22.54 b	1010.00 ± 62.86 a
PT (°C)	87.02 ± 0.25 b	87.40 ± 0.22 ab	86.43 ± 0.08 c	87.71 ± 0.37 a

Note: PV, peak viscosity; TV, trough viscosity; BD, breakdown; FV, final viscosity; SB, setback; PT, pasting temperature. Viscosity parameters are expressed in centipoise (cP), and PT is expressed in °C. Values are presented as mean ± standard deviation (SD). Different lowercase letters within the same trait indicate significant differences among cultivars (*p* < 0.05).

## Data Availability

The original contributions presented in this study are included in the article and its Supplementary Materials. Further inquiries can be directed to the corresponding authors.

## Supplementary Materials

The following supporting information can be downloaded at: https://www.mdpi.com/article/10.3390/foods15183340/s1, Figure S1: Number-based starch granule-size distributions of four wheat cultivars at 23 and 29 DAA and maturity; Figure S2: XRD patterns of starch from four wheat cultivars at 23 and 29 DAA and maturity; Figure S3: DSC thermograms of starch from four wheat cultivars at 23 and 29 DAA and maturity; Table S1: Two-way ANOVA of the effects of cultivar, year, and their interaction on wheat grain and flour quality traits; Table S2: Effects of cultivar, developmental stage, and cultivar × stage interaction on grain weight, moisture content, and morphological traits during wheat grain filling; Table S3: Effects of cultivar, developmental stage, and cultivar × stage interaction on starch content and protein composition during wheat grain development; Table S4: Two-way ANOVA of the effects of cultivar, developmental stage, and their interaction on starch granule size distribution and physicochemical properties.
