# Supplementary material for: Protein Composition and Starch Structural Differentiation During Wheat Grain Filling Are Associated with Mature Grain Quality
Source: Foods. 2026 Sep 20;15(18):3340. doi: 10.3390/foods15183340 (PMC13606141; doi:10.3390/foods15183340)
Supplement: Supplementary file 1 [file foods-15-03340-s001.zip › Supplementary Figures.pdf]

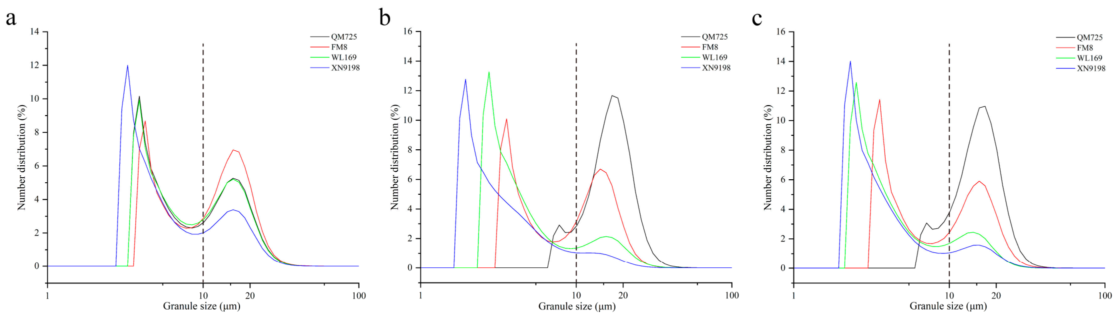

Figure S1. Number-based starch granule-size distributions of four wheat cultivars at 23 and 29 DAA and maturity. (a) 23 DAA; (b) 29 DAA; (c) maturity. Curves show the number-based particle-size distributions of starch granules from QM725, FM8, WL169, and XN9198. The vertical dashed line at 10 μm indicates the threshold used to distinguish B-type (≤10 μm) from A-type (>10 μm) starch granules.

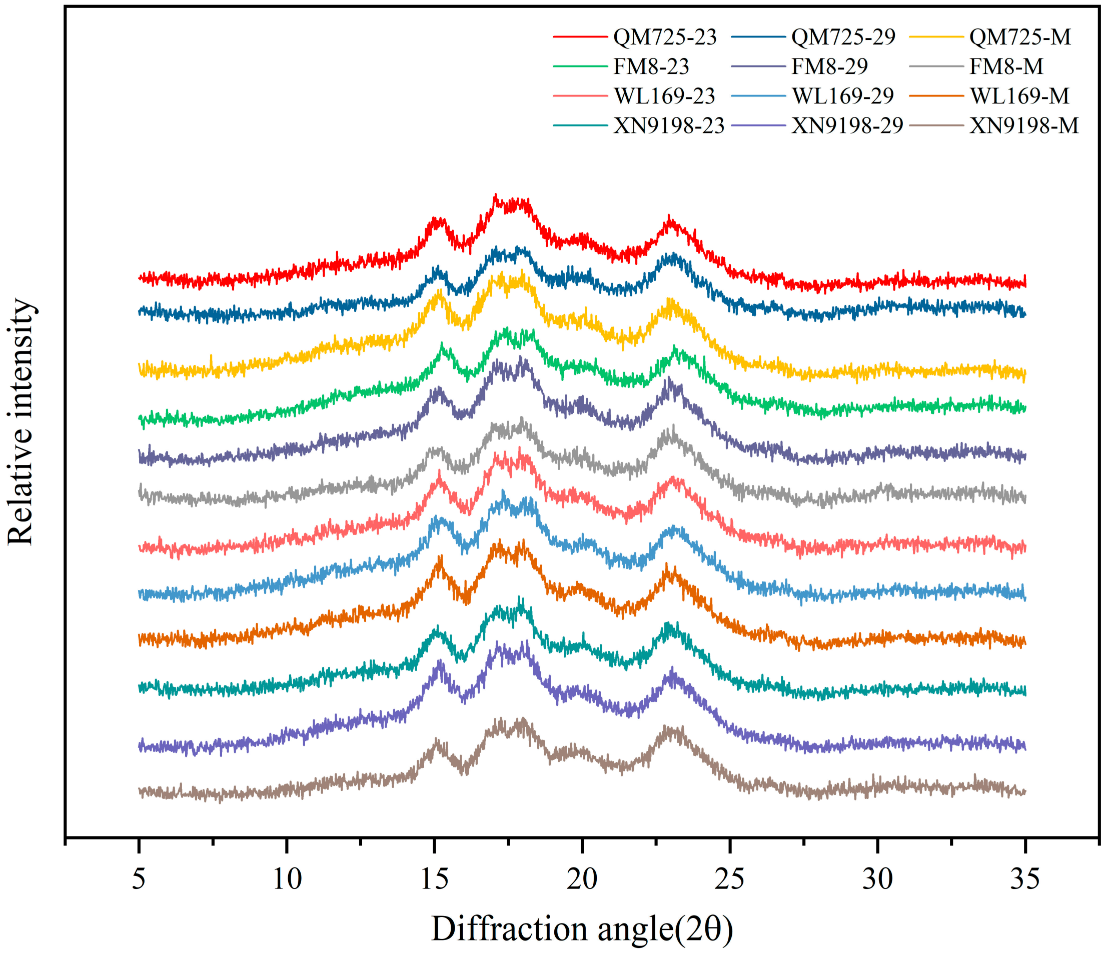

Figure S2. XRD patterns of starch from four wheat cultivars at 23 and 29 DAA and maturity. QM725, FM8, WL169, and XN9198 were analyzed at 23 and 29 DAA and at maturity. The diffraction patterns were vertically offset for clarity.

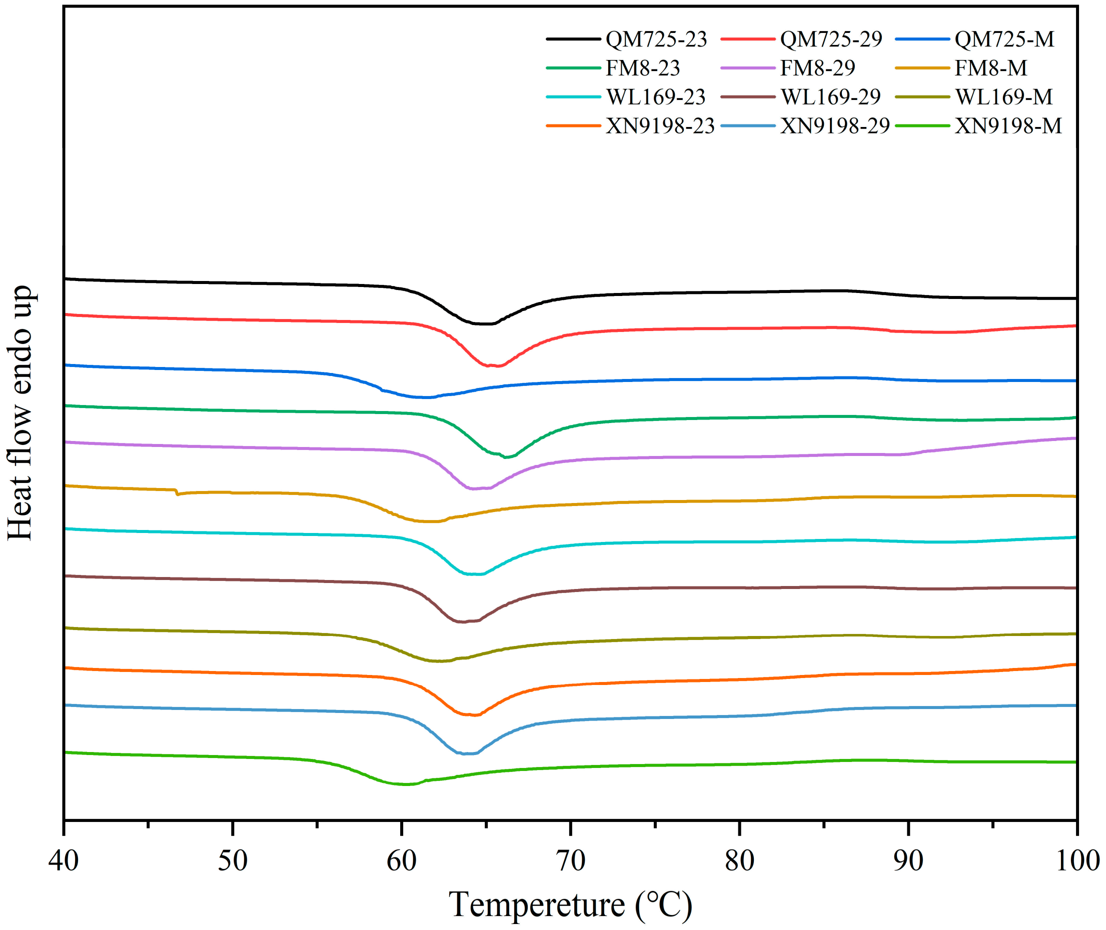

Figure S3. DSC thermograms of starch from four wheat cultivars at 23 and 29 DAA and maturity. Curves were vertically offset for clarity; endothermic heat flow is shown upward.
